# Asynchronous Federated Learning for Improved Cardiovascular Disease Prediction Using Artificial Intelligence

**DOI:** 10.3390/diagnostics13142340

**Published:** 2023-07-11

**Authors:** Muhammad Amir Khan, Musleh Alsulami, Muhammad Mateen Yaqoob, Deafallah Alsadie, Abdul Khader Jilani Saudagar, Mohammed AlKhathami, Umar Farooq Khattak

**Affiliations:** 1 Department of Computer Science, COMSATS University Islamabad Abbottabad Campus, Abbottabad 22060, Pakistan; amirkhan@cuiatd.edu.pk (M.A.K.); mateen@cuiatd.edu.pk (M.M.Y.); 2 Information Systems Department, Umm Al-Qura University, Makkah 21961, Saudi Arabia; dbsadie@uqu.edu.sa; 3 Information Systems Department, College of Computer and Information Sciences, Imam Mohammad Ibn Saud Islamic University (IMSIU), Riyadh 11432, Saudi Arabia; aksaudagar@imamu.edu.sa (A.K.J.S.); maalkhathami@imamu.edu.sa (M.A.); 4 School of Information Technology, UNITAR International University, Kelana Jaya, Petaling Jaya 47301, Selangor, Malaysia

**Keywords:** heart disease prediction, machine learning, reliable deep models, healthcare applications, distributed machine learning

## Abstract

Healthcare professionals consider predicting heart disease an essential task and deep learning has proven to be a promising approach for achieving this goal. This research paper introduces a novel method called the asynchronous federated deep learning approach for cardiac prediction (AFLCP), which combines a heart disease dataset and deep neural networks (DNNs) with an asynchronous learning technique. The proposed approach employs a method for asynchronously updating the parameters of DNNs and incorporates a temporally weighted aggregation technique to enhance the accuracy and convergence of the central model. To evaluate the effectiveness of the proposed AFLCP method, two datasets with various DNN architectures are tested, and the results demonstrate that the AFLCP approach outperforms the baseline method in terms of both communication cost and model accuracy.

## 1. Introduction

Data in the healthcare industry are generally dispersed because of the complex nature of the systems and procedures involved in providing healthcare. For instance, various hospitals may only be able to view the clinical information related to the patients who are treated at that hospital. These records include protected health information (PHI), which is very sensitive information [1]. From that dataset, we will obtain various insights that help us know the weights of each feature, as well as how the features are interrelated. However, this time, the only goal is to determine the probability of a person being affected by a savior heart problem.

There is a significant opportunity for artificial intelligence to be used in medical applications, particularly ones that aim to enhance medical services for individuals as well as medical institutions. Breathing difficulties, bodily heaviness, and foot edema are typical signs of cardiovascular disease. Due to the accuracy and execution time limits of present diagnostic procedures, researchers are continuously looking for effective strategies for the early diagnosis of cardiac disease. These training data were collected from a wide variety of clinical observations, including, but not limited to biological sensors, individual patients, clinical institutions, hospitals, pharmaceutical enterprises, and health insurance organizations [2]. In a learning technique that is asynchronous, the clients and a temporarily weighted aggregation of the local models are performed on the server [3].

**Figure 1:**
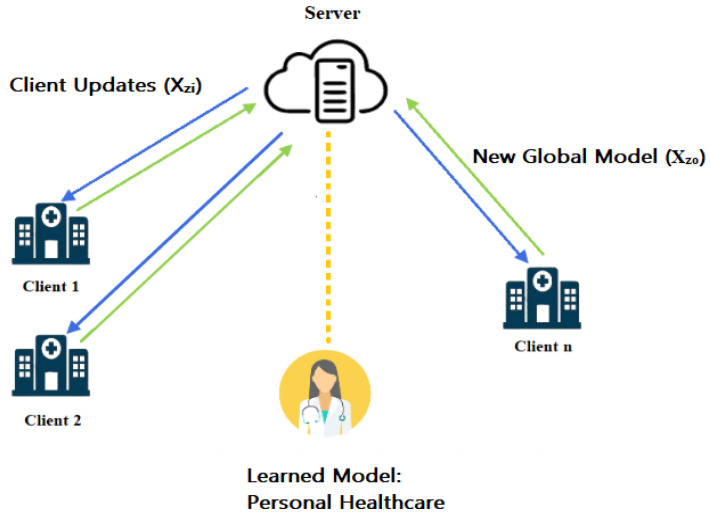
Federated learning in a healthcare environment.

The process of obtaining and analyzing such data is subject to strict rules, one example of which is the Health Insurance Portability and Accountability Act (HIPAA) which was designed to protect patient privacy. This is a significant obstacle for contemporary data mining and machine learning (ML) technologies, such as deep learning, which often needs a large quantity of data to be used for training purposes. Federated learning (FL) is a relatively new method that has emerged in recent years as a response to the challenge of training a strong deep learning model using federated medical data while maintaining patient confidentiality [4,5]. This technique allows the training of decentralized federated learning models without the need for transferring medical data via a coordinated central aggregated server. A typical working schematic of FL in a healthcare environment is depicted in Figure 1. The deep learning models used by medical institutions function as client nodes, which train the model locally before occasionally sending them to an aggregate server for further processing. After coordinating and aggregating the local models contributed by each node to produce a global model, the central server then sends the global model to all the other nodes in the network. During the process of training, the training data are never sent and are instead kept secret by each node. This is an important point to keep in mind that only the model’s weight and certain parameters are sent across, protecting the privacy of any medical information that may have been collected. As a result of these factors, FL can alleviate a significant number of security worries since it maintains sensitive and confidential data [6].

When analyzing enormous volumes of difficult medical data, researchers employed a range of data mining and federated learning methodologies. The medical staff was able to make more accurate diagnoses of heart disease with the assistance of this paper. This provided a model that was based on supervised learning strategies including decision trees, the k-nearest neighbor, naive Bayes, and random forest (RF) algorithms. In addition to this, the paper included a wide range of factors that were connected to cardiovascular disease. It did so by using a dataset that was already in existence and which was taken from the UCI repository of persons who were suffering from cardiac diseases. The dataset had a total of 303 distinct cases and 76 different types of attributes. Only 14 of these 76 characteristics were considered for the testing process; these were the characteristics that were thought to be important for proving the performance of different algorithms. That study’s objective was to arrive at an estimate of the participants’ future risk of developing coronary artery disease (CAD), which served as the basis for the research. According to the results, it seemed as if the KNN algorithm achieved the highest possible degree of accuracy [7].

In previous research, an innovative method for assessing crucial outcomes by making use of AI techniques to increase the accuracy of our predictions for cardiovascular infections was proposed. Employing the technique for federated learning known as logistic regression boosted the level of execution while maintaining an accuracy level of 89% when predicting coronary disease [8].

In this paper, we will be working closely with the prediction of heart diseases by looking into the heart disease dataset. Many layers of deep neural networks (DNNs) are separated into deep and shallow levels using the asynchronous learning technique. The asynchronous learning strategy updates the parameters of the deep layers less often than those of the shallow levels. In addition to this, a temporally weighted aggregation method is used on the server to make use of the locally trained models that have been stored there before. This helps to improve the accuracy and convergence of the central model. The suggested technique was tested experimentally on two datasets equipped with a variety of DNNs. According to the findings that we obtained, the asynchronous federated deep learning outperforms the baseline approach in terms of both the cost of communication and the accuracy of the model. The asynchronous federated learning (Async-FL) technique that was developed can achieve high classification efficiency while also assuring privacy and flexibility, and limiting the amount of network bandwidth that is used. Our vital contributions to this research are listed below.

- Exploring synchronous and asynchronous communication;
- Providing a broad overview of distributed federated learning techniques;
- Proposed an asynchronous FL cardiac prediction (AFLCP);
- Finally, comparing the accuracy and importance of the loss function between synchronous and asynchronous federated learning.

The remaining parts of the article are structured as described in the following paragraphs. The relevant research on using asynchronous federated learning to treat heart diseases are surveyed in Section 2. Section 3 describes the official issue along with the dataset and provides our suggested asynchronously updating federated learning algorithm and our regarded asynchronously updating federated learning-based system paradigm. In Section 4, the effectiveness of our proposal is assessed through comparisons.

## 2. Related Work

The first framework for federated learning was established in [9], and from the results of their experiments, it was determined that federated learning methods are not appropriate for this environment. Structured updates and sketched updates, both of which make use of data compression and reconstruction techniques, are the two methods the authors of [10] believe might be used to cut down on the costs of uplink transmission. Federated averaging (FedAVG) is a newer kind of federated learning. This type of federated learning was created to acquire a centralized prediction model of Google’s Gboard software, and it may be incorporated into a mobile phone to safeguard the user’s privacy.

Monitoring systems for electrocardiograms (ECGs) that are hosted in the cloud make use of a variety of methodologies, including feature extraction and categorization. DWT and ANN identified binary heartbeats [11]. CNNs categorized 12 cardiac rhythms using single-lead ECG [12]. Observing an ECG over time using DWT and nonlinear DDE-based optimization cannot infer system models under varying cardiac conditions. There is a lot of work to be conducted on the classification structure of these algorithms. In research [13], a genetic algorithm (GA) was used to choose the most suitable DDE-based classification model. In [14], a sparse decomposition was used to effectively extract features, and classification techniques such as k-nearest neighbor (KNN), support vector federated (SVM), and radial basis function neural network were applied. From 12-lead ECGs, different manual feature extraction methodologies were applied to obtain parameters such as the P-wave interval, QRS interval, and QT interval. In [15], the support vector federated model was utilized to detect myocardial infarction. However, the majority of the existing body of research does not focus on the development of lightweight AI models; rather, it employs a centralized federated learning algorithm to ignore the privacy breaches that occur throughout the process of data collection. This is the case because lightweight AI models require less computing power. Because of this, it is necessary to gather and exchange private ECG data to develop a data-driven federated learning model to identify heart disease occurrences.

As a result, it is impossible to implement these strategies in nodes owing to the extremely high computational cost involved in doing so. A few different pieces of research looked at ECG analytics that were centered on edge computing. In [16], the researcher designed an ECG analysis method and implemented it on an IoT-based embedded platform. The technique included noise-filtering and manual feature extraction stages. In [17], the researcher proposed a federated learning-based distributed algorithm that makes it possible for each medical institution to participate in the training of the AI model in a locally cooperative manner. This was performed to bring attention to the need for collaborative online learning. However, the practicality of the decentralized online federated learning approach for deployment at the nodes for remote ECG monitoring has not yet been examined in detail in the current literature; as a result, the emphasis of this research is on filling this research gap.

Asynchronously updated FL architecture (Async-FL) for mobile and deployable nodes enables decentralized and collaborative arrhythmia diagnosis without cloud ECG data interchange. Raw single-lead ECG data caused dispersed FL topologies, patient data privacy, and network overhead. In rigorous testing, Async-FL discovered arrhythmias with reduced memory and execution time. This reduced network overhead for more nodes. This Async-FL ECG monitoring use case may help build the next generation of smart and remote health monitoring system at scale when pandemics such as the new coronavirus increase demand.

Heart disease prediction was suggested using a privacy-aware decentralized federated learning framework. FedMA and M-ABC optimized cardiac illness prediction and healthcare privacy. This enhanced cardiac diagnosis, training, and communication. To test the system, model prediction-based parameters and communication efficiency using baseline federated learning FedAvg, FedMA, and FedMA utilizing PSO optimizer algorithms were analyzed. The framework increased classification error, accuracy, sensitivity, and communication efficiency. The framework has 2.6% greater accuracy, 7% less classification error, 1.8% more precision, 7.1% higher sensitivity, and 12% fewer rounds to obtain maximum accuracy. The model’s learning rate influenced IoMT client site scalability. Other feature selection and optimization strategies helped privacy-aware healthcare forecasting [18].

Federated learning executes the process of updating the global model in a synchronous way. This means that the FL server waits for a certain number of local models to be submitted from distributed devices before calculating and distributing a new global model. We propose something called asynchronous federated learning (Async-FL). This system enables each client to continually upload its model depending on its capabilities and the FL server will be used to decide when to asynchronously update and broadcast the global model [19]. A methodology for smart healthcare systems’ predictive evaluation based on deep learning is proposed in [20]. However, possible drawbacks, such as the requirement for labeled data and biases in training data, are not considered. The authors of [21] use an iterative reconstruction technique to address the problem of lowering the radiation exposure in CT images. This method provides a solution; however, there may be issues with computational complexity and newly produced artifacts that need to be discussed. In [22], a new sparse decomposition-based picture fusion technique is described. The method’s sensitivity to noise and the probable loss of minute features during fusion are a couple of them. The authors in [23] give an overview of pulse-coupled neural networks used in image processing. Limitations such as parameter tuning difficulty and specialized hardware needs should be looked at while considering their applications and developments.

For tackling the issues of asynchronous communication and data imbalance in distant healthcare systems, authors in [24,25,26] offer federated machine learning. The authors suggest employing federated learning in an asynchronous and weighted manner to increase the precision of disease detection. The research highlights the potential of federated learning in enhancing diagnostic outcomes while offering a creative response to privacy issues in healthcare.

## 3. Materials and Method

Machine learning models are frequently evaluated using measures including accuracy, precision, and F1-score [27,28,29]. Each metric can be explained as follows:

- **Accuracy:** By assessing the percentage of cases that were properly classified out of all occurrences, accuracy measures the general correctness of the model’s predictions. In terms of accurately detecting both positive and negative cases, it gives a sign of how effectively the model performs.
- **Precision:** The ability of the model to properly identify positive instances among all instances predicted to be positive is measured by precision. Indicating the percentage of real positives among all instances anticipated as positive, it focuses on how accurate positive predictions are. When the cost of false positives is significant, precision is advantageous because it ensures a low rate of misclassifying negative instances as positive.
- **F1-score:** Harmonic mean of recall and precision is the F1-score. It provides a fair assessment of the model’s performance by combining precision and recall into a single statistic. When the dataset is unbalanced or when both false positives and false negatives need to be reduced, it is especially helpful. An increase in the F1-score from 0 to 1 indicates greater overall performance.

Infected people are more likely to have cardiac arrest, and this is particularly true of those in the most vulnerable populations [30]. Monitoring cardiac activity in a decentralized fashion is required because of the frequently shifting COVID-19 stresses and their influence on heart health. Nodes are necessary to cover subjects in places with limited access to healthcare for long-term cardiac monitoring. Each node may be used to determine the cardiac status (irregular heartbeats) of a patient. Several nodes may be used to condense information about a certain area, giving healthcare practitioners a more complete picture of the area.

As a result, the first obstacle is to speed up online and collaborative learning across nodes so that each node may adjust to and improve upon itself considering the diverse ways in which it acquires and uses data. In addition, there is a high bar for protecting users’ privacy since many are wary of entrusting their sensitive health information to a server in the cloud. In addition, after the local automated decision-making is complete for each node, the private data should be safely employed without communicating raw data elsewhere and then erased. As a result, the nodes need a distributed learning architecture to enable the secure and efficient extraction of unique ECG characteristics. Due to the nodes’ lack of universal interest, the job of decentralized or distributed collaborative learning may pose significant difficulties. The proposed system has looked at many forms of federated learning, such as the more common synchronous form and the more flexible asynchronous form, to find a solution to this problem. In this scenario, nodes will be the edge users, and they will be able to communicate private data knowledge with the server to acquire access to global information and guarantee online learning. This method will protect users’ privacy, speed up processing, and adjust to a wide range of data distribution patterns. When the server has received all the nodes’ local models, it will update the global model to make it more accurate and efficient in heart disease prediction. To rephrase, the goal of loss function reduction is as follows:

(1) $$\mathrm{Min}\;\sum_{i=1}^{n}\frac{A_{i}}{A}f_{i}(\omega )$$

where $f_{i}$(ω) represents the cloud’s loss function, $A_{i}$ represents nodes’ private ECG sample data, and A represents ECG data utilized by the cloud for training. The artificial intelligence-based model’s parameters for a certain area are shown by the weight vector (ω) for that node. For a certain amount of prediction error, the value of the loss function will go up. Thus, for learning convergence, which happens during the information exchange iterations of the learning phase, it is important to make sure that the local AI model’s parameters are always the same after receiving the updated model from the server. Even if the nodes and the server don’t share raw ECG data, they can still use the global model to make decisions locally.

The main goal of the research is to make a system that allows each node to train a decentralized global model to update its local model using its own private raw ECG data. Each node can send its updated local model to other nodes and the cloud server so that global model changes can happen at different times. This interactive learning process will go on if the loss function is not lowered, and the accuracy of the global model meets a performance criterion. Thus, the main goal of this research paper is to make an asynchronous, decentralized architecture for learning that can find heart diseases, keep data safe, and cut down on network load.

### 3.1. Dataset Descriptions

The two datasets DS1 and DS2 were taken from the machine learning library at UCI and Switzerland. It consists of 16 properties, the most important of which is the ‘target’ attribute, which indicates whether a person has heart disease or not. This collection is comprised of 303 records and 76 characteristics in total. Table 1 provides an example of the DS1 and DS2 datasets. The datasets include both continuous and categorical values in its arrangement of data. To ensure that the datasets can be utilized as input into the machine learning model, appropriate pre-processing procedures need to be applied to it. If the target column has a value of one, it indicates that the individual under observation has heart disease, whereas a value of zero suggests that they do not have heart disease. The remaining data, minus 33% of it, are utilized for training purposes. The remaining data are held out as training data. Using the TensorFlow Federated framework, the datasets are transformed into federated datasets. It is estimated that there will be a total of five clients. About 40 records are sent to each client. The testing will be conducted using the remaining data.

### 3.2. Proposed Method

In this section, the specialized lightweight CNN model aids in the development of global and local AI models in the cloud and nodes, respectively. In previous work [31], a deep learning model for the centralized prediction of heart diseases at the nodes was proposed; we refer to this model as deep learning-based lightweight heart disease predictions. The single-lead raw ECG heartbeat is fed into the lightweight AI model that will be installed at the nodes for analysis of ECG as *M* = {*m*1*, m*2*, m*3*… mn*}, and the predicted class labels are output as *N* = {*n*1*, n*2*, n*3*… nk*}. Whether the current pulse falls within the kth class is indicated by the value of *nk,* which in this case takes the values 0 and 1. The classification decision is performed using the radial basis-inspired support vector machine (R-SVM) classifier [32]. The decision for classification is computed using Equation (2) below.

(2) $$D\;(f_{d})=\sum_{d=1}^{n}\gamma j.\;R_{F}\left(d,\;d_{j}\right)+m_{r}$$

where the kernel function of the standard SVM is modified using the radial basis *R*_F_ (d, d_j_) = *e*^(−γ|[d − dj]|^2^)^. The input dataset is represented by d, the weight is represented as γj, and the margin is *m_r_*.

#### Proposed Algorithm: Asynchronous FL Cardiac Prediction (AFLCP)

The server-side algorithm is a representation of the algorithm that is carried out on the distant cloud server to update the global model. This algorithm takes the collection of local model parameters or weights of all nodes as input, which are represented as ω, and in output, it gives the most recent version of the global model (X_g_). The working of our proposed method is described as follows. Table 2 represents the description of symbols used in the proposed method.

Stap 1. E is initialized with the minimum loss threshold.
Stap 2. X_g_ is initialized with the existing global model.
Stap 3. It involves accessing the learning performance of the model, and at each iteration, the performance is compared with the loss threshold (E) that was established in an earlier step. Because of this, the entire learning process will continue to take place at the distant cloud server for as long as the current loss value is more than E.
  a. Aggregating local model parameter updates Xg and loss evaluation to ω.
Stap 4. End of iterations.
Stap 5. X_g_ value is updated.

**Figure 2:**
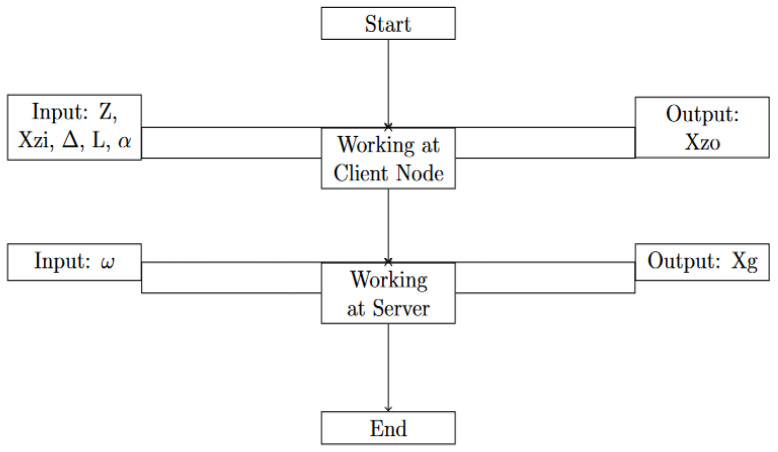
The working flowchart of our proposed method.

The client-side algorithm is a representation of the algorithm that is executed on the side of the nodes. This algorithm takes the method into consideration that operates on a single node. The algorithm begins with inputs; the specifics of these inputs are Z, X_zi_, Δ, L, α. The Algorithm 1 and flowchart depicted in Figure 2 present the working of our proposed method and the detail of each step is described as follows.

Stap 1. The originally trained AI model (Xzi) is applied to the local model of Z nodes.
Stap 2. Initialization of the data size of the threshold to its default value in preparation for the subsequent phases.
Stap 3. Initialization of timestep (γz) and block (Bz) to their default values in preparation for the subsequent phases.
Stap 4. The new private data (Mz) are loaded, and the size of the data is determined by comparing it with the threshold (β) data size.
Stap 5. The classification decision is computed using the R-SVM classifier.
Stap 6. The iteration begins from 1 to L. L is the number of iterations that occur when the whole procedure is being carried out at nodes.
  i. If the condition [(ℓ mod Δ) = 0] is satisfied, then it will execute the body of an if statement.
    a. When the condition is met, which occurs after iteration, the time ℓ that was calculated is saved in the time-step (γ_z_) list.
    b. The local weights (ω_z_) are extracted from the initial model together with α. Here, α denotes the deep parameter exchange ratio. α indicates the deep parameter ratio that is contributing to the deep exchange rate. The information on the iteration that takes place during the deep exchange with the cloud is stored inside the timestep γ parameter.
    c. The extracted weights (ω_z_), timestep (γ_z_), and block (B_z_) are transferred to the cloud server.
  ii. If the condition [(ℓ mod Δ) = 0] is not satisfied, then it will execute the body of the else statement.
    a. The shallow parameters of the (1 − α) ratio taken from the local model (Xzi) are stored in ωz.
    b. The extracted weights (ωz) are passed to the cloud server.
  iii. End of if–else statement.
Stap 7. The local model of Z nodes is updated, which makes use of the aggregated model that was retrieved from the server.
Stap 8. All the global model states and information pertaining to timestep ℓ are saved in the Bz so that they may be accessed later.
Stap 9. The loop ends.
Stap 10. Once the training has been completed for L iterations, the used data M are deleted from the cache in a permanent manner to improve the safety of the user’s data.

**Algorithm 1:** Asynchronous Cardiac Prediction (ACP)**Input**: Z, X_zi_, Δ, L, α **Output**: X_zo_ **Working at Client Node**1.X_zi_ ← initially obtained model from server.2.β ← initialize data size threshold.3.Initialize γ_z_ and B_z._4.M_z_ ← new data obtained and evaluate M_z_’s size by comparing with β.5.Classification decision is performed using Equation (2).6.for ℓ= 1 to L
i.if (ℓ mod Δ) = 0 then
a.ℓ = γ_z_b.ω_z_ ← α’s local weight extracted from X_zi_c.Pass ω_z_, B_z_, γ_z_ to the server.
ii.else
a.ω_z_ ← (1 − α)’s extract local weights from X_zi_b.Pass ω_z_ to the server.iii.End.
7.X_zo_ ← send updated model to server.8.B_z_ ← save ℓ time global model state and data access information.9.End.10.Delete M_z_ from storage.**Working at Server** **Input:** ω
**Output:** X_g_
1.E ← minimum loss threshold.2.X_g_ ← existing global model.3.While (currloss > E) do
a.ω ←Aggregating local model parameter updates X_g_ and loss evaluation.
4.End.5.return X_g._



## 4. Experimental Results and Discussion

In this section, we will examine how the proposed technique is performed and the datasets used to assess its efficacy. We will also compare the performance of the proposed technique. We utilized the random forest and grid search as suggested by the researchers in [33,34,35,36,37]. Table 3 shows the effect of increasing the number of nodes on the accuracy, precision, and f1-score for the synchronous and asynchronous federated learning methods. For a higher number of clients, our proposed asynchronous approach achieves better performance because the weights from clients are aggregated asynchronously and this results in better performance of the model. To test and verify the effectiveness of the proposed method, we implemented the experimentation in the *TensorFlow Federated (TFF)* library on Intel ^®^ Core i7 with 16 GB RAM. For implementation, we faced the challenge of dataset distribution among the client nodes. To overcome this challenge, we distributed the dataset equally among the clients. We compared the performance of the proposed method with the existing state-of-the-art FL-Avg method.

**Figure 3:**
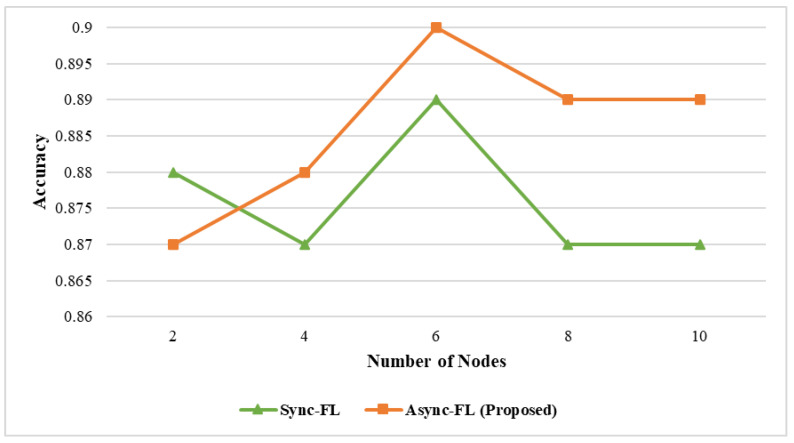
Effect of accuracy and number of nodes on dataset DS1.

**Figure 4:**
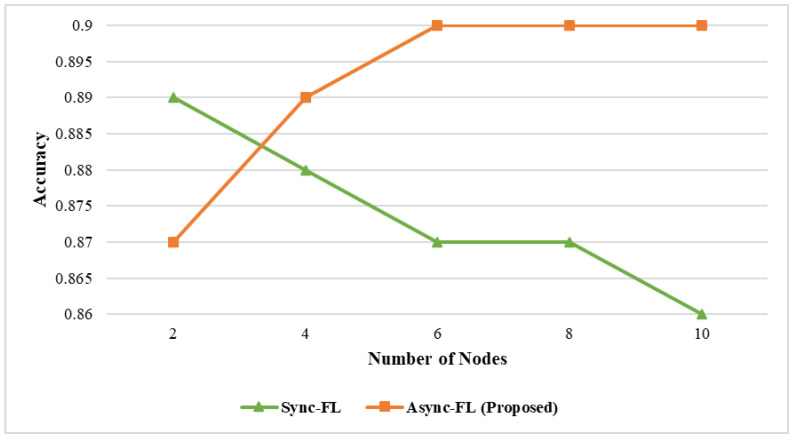
Effect of accuracy and number of nodes on dataset DS2.

**Figure 5:**
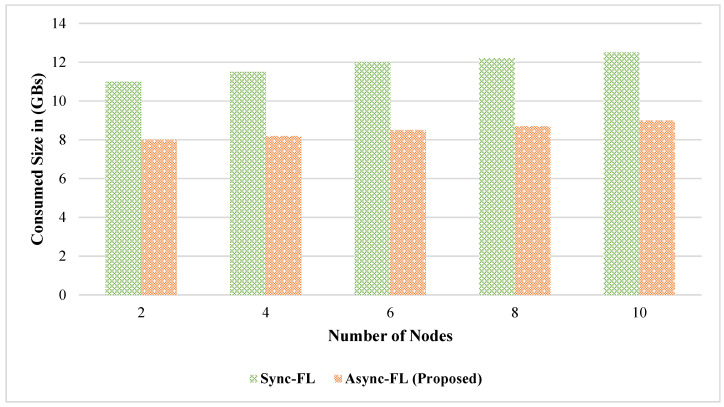
Comparison of memory consumption on DS1.

**Figure 6:**
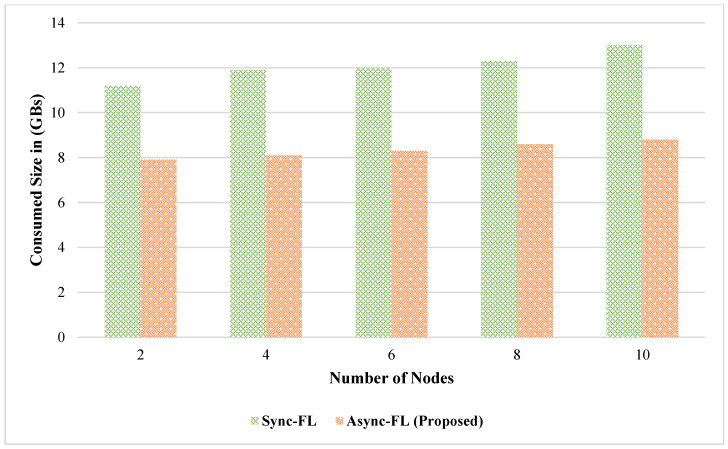
Comparison of memory consumption on DS2.

The effect of learning accuracy for DS1 and DS2 datasets is shown in Figure 3 and Figure 4. Our proposed framework utilizes a better global and client model for weight aggregation and decision classification which enables us to achieve a higher learning accuracy on both datasets. The comparison of memory utilization is depicted in Figure 5 and Figure 6.

**Figure 7:**
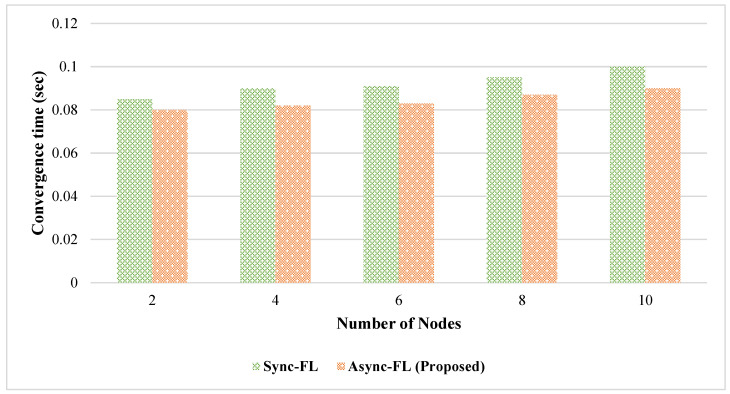
Comparison of algorithm convergence rate for dataset DS1.

**Figure 8:**
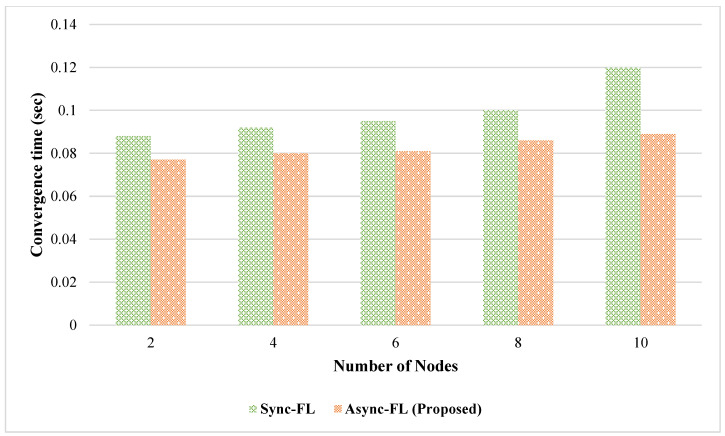
Comparison of algorithm convergence rate for dataset DS2.

Figure 7 and Figure 8 show the time required by the number of client nodes to converge. Our proposed asynchronous approach shows better convergence of the algorithm for a higher number of client nodes on both datasets because the client updates are aggregated at the global cloud end asynchronously.

## 5. Conclusions

This paper has presented a privacy-aware approach for predicting heart diseases using the heart disease dataset with an asynchronous federated learning technique. The proposed technique updates the parameters of deep and shallow levels of the DNNs asynchronously and utilizes a temporally weighted aggregation method to improve the accuracy and convergence of the central model. Experimental results on two datasets have shown that the proposed asynchronous federated deep learning approach outperforms the baseline approach in terms of communication cost and model accuracy. The contributions of this research include exploring synchronous and asynchronous communication, providing an overview of distributed federated learning techniques, proposing an asynchronous federated learning approach for cardiac prediction, and comparing the performance of synchronous and asynchronous federated learning in terms of accuracy and loss function value. The proposed technique offers a privacy-preserving, flexible, and efficient solution for cardiac prediction with limited network bandwidth usage. This study opens up new avenues for future research in the field of distributed machine learning techniques for healthcare applications. The limitation of our work is that we have not tested the effectiveness of the scalability issue. The rehabilitation and treatment of several more severe illnesses, such as Parkinson’s, diabetes, liver cancer, skin cancer, and breast cancer, will be our primary emphasis in the future.

## Figures and Tables

**Table 1:** Information of each attribute to predict heart diseases.

S. No	Attributes	Value
1	Age in years	>79 = 2, 61–79 = 1, 51–60 = 0, 35–50 = −1, <35 = −2
2	Sex	Female = 0, Male = 1
3	Chest Pain Type (4 different values)	0–0.3 = -1, 0.9–1.2 = 0, 1.8–2.1 = 1, 2.7–3.0 = 2
4	Resting BP	Above 139 mmHg = High = 1 120–139 mmHg = Normal = 0 Below 120 mmHg = Low = −1
5	Serum Cholesterol	>240 mg/dL = High = 1 200–239 mg/dL = Normal = 0 <200 mg/dL = Low = −1
6	Fasting blood sugar>120mg/dl (Boolean)	True = 1 False = 0
7	Resting electrocardiographic result (3 values)	Hypertrophy = 2 ST T = 1 Normal = 0
8	Diabetes	Yes = 1 No = 0
9	Exercise-induced angina	Yes = 1 No = 0
10	ST depression induced by exercise	Up = 2 Flat = 1 Down = 0
11	Slope of peak exercise ST segment	<0.5 mm = Normal = 0 >0.5 mm = High = 1
12	Smoke	Yes = 1 No = 0
13	Status of heart (3 possible values)	Reversible defect = 7, Normal = 3, fixed defect = 6
14	Heart disease (target)	Yes = 1 No = 0
15	Number of major vessels colored by fluoroscopy	Vessel 0 = 0 vessel 1 = 1 vessel 2 = 2 vessel 3 = 3
16	Maximum heart rate	<69 bpm = Low = −1 70–90 bpm = Normal = 0 >91 bpm = High = 1

**Table 2:** Table of Symbols.

Symbol	Description
X_zi_	Initial model for node Z
X_zo_	Output local model from node Z
Δ	Iteration time
L	Total iterations
α	Deep parameter exchange ratio
B	Block
β	Minimum acceptable size of ECG
γ	Timestep
ℓ	Iteration
Z	Number of nodes
ω_z_	Local weights extracted from X_zi_
M_z_	New data obtained
ω	Global weight
X_g_	Existing global model
E	Minimum loss threshold

**Table 3:** Proposed method with varying nodes classification performance on two test datasets.

Method	Datasets	Metrices	Number of Nodes				
			2	4	6	8	10
Sync-FL	DS1	Accuracy	0.888	0.889	0.889	0.878	0.877
		Precision	0.878	0.879	0.874	0.868	0.871
		F1-Score	0.880	0.882	0.881	0.872	0.872
	DS2	Accuracy	0.893	0.889	0.886	0.895	0.863
		Precision	0.885	0.868	0.859	0.881	0.838
		F1-Score	0.867	0.862	0.867	0.865	0.849
Async-FL	DS1	Accuracy	0.869	0.879	0.891	0.879	0.878
		Precision	0.873	0.878	0.883	0.875	0.877
		F1-Score	0.869	0.876	0.887	0.875	0.875
	DS2	Accuracy	0.871	0.886	0.895	0.895	0.899
		Precision	0.841	0.856	0.889	0.881	0.891
		F1-Score	0.852	0.868	0.867	0.869	0.874

## Data Availability

We ran simulations to see how well the proposed approach performed. Any questions concerning the study in this publication are welcome and can be directed to the lead author (Muhammad Amir Khan) upon request.
